# Supplementary material for: The Giardial Arginine Deiminase Participates in Giardia-Host Immunomodulation in a Structure-Dependent Fashion via Toll-like Receptors
Source: Int J Mol Sci. 2022 Sep 30;23(19):11552. doi: 10.3390/ijms231911552 (PMC9569872; doi:10.3390/ijms231911552)
Supplement: Supplementary file 1 [file ijms-23-11552-s001.zip › ijms-1902008-supplementary.pdf]

The giardial arginine deiminase participates in *Giardia*-host immunomodulation in a structure-dependent fashion via Toll-Like Receptors.

Fernández-Lainez, F., *et al.*

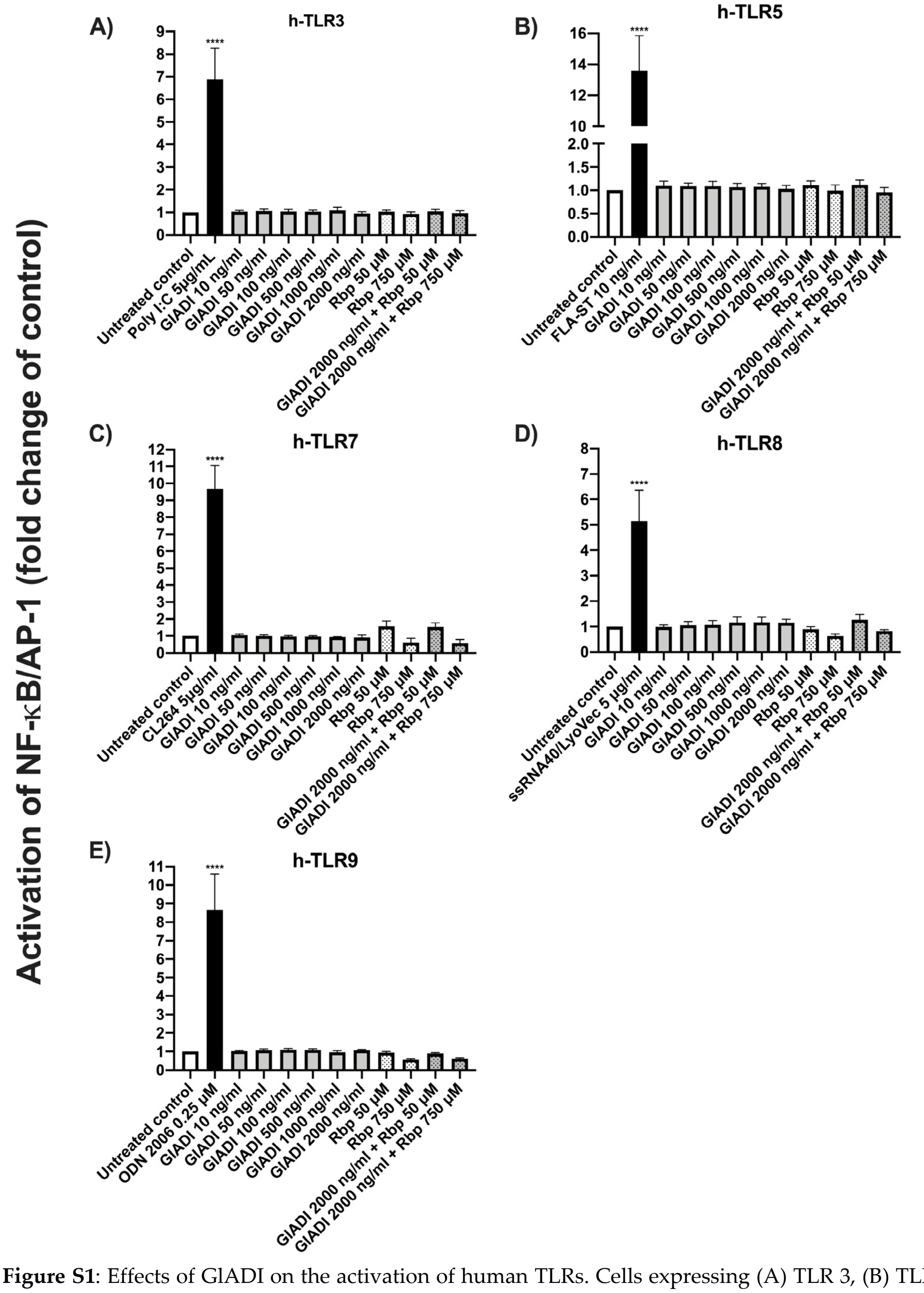

Figure S1: Effects of GlADI on the activation of human TLRs. Cells expressing (A) TLR 3, (B) TLR5, (C) TLR7, (D) TLR8, and (E) TLR9 were incubated for 24h with rising concentrations of GlADI, Rabeprazole (Rbp), or their combination. Afterward, NF-ΚB/AP-1 release was determined.
